# Air Pollution Intervention: Study Links Use of Face Masks to Improved Cardiovascular Outcomes

**DOI:** 10.1289/ehp.120-a122b

**Published:** 2012-03-01

**Authors:** Julia R. Barrett

**Affiliations:** Julia R. Barrett, MS, ELS, a Madison, WI–based science writer and editor, has written for *EHP* since 1996. She is a member of the National Association of Science Writers and the Board of Editors in the Life Sciences.

Air pollution from traffic and industrial sources increases cardiovascular morbidity and mortality, especially in populations with underlying cardiovascular disease. Environmental interventions to reduce pollution would be ideal, but their implementation may be hampered in countries where emissions reductions are trumped by economic growth. A new study explores face masks as a simple and practical intervention for reducing individuals’ exposure to particulate air pollution and finds their use improves several cardiovascular health measures in people with coronary heart disease [*EHP* 120(3):367–372; Langrish et al.].

Vehicle traffic and industry emit a variety of toxicants, including particulate matter, carbon monoxide, sulfur dioxide, and nitrogen dioxide. Previous studies have shown that inhaling combustion emissions may increase blood pressure and adversely affect vascular and cardiac function, plausibly explaining the detrimental cardiovascular effects associated with breathing heavily polluted urban air.

In the current study, nonsmoking patients with a history of coronary heart disease were recruited in March 2009 at 2 Beijing hospitals. After participants’ health and physical condition were assessed, they completed 2-hour walks around Beijing’s city center on 2 separate days. Participants were randomly assigned to wear a highly efficient air-filtering mask on 1 of the 2 walks. The mask was worn outdoors and during much of the time indoors for 24 hours before the study day as well as the entire study day itself. On study days, participants also wore equipment to monitor their air pollutant exposure as well as blood pressure and heart function. They completed questionnaires on their physical symptoms, their perceptions of pollution levels and exertion, and the tolerability of the face mask.

**Figure:**
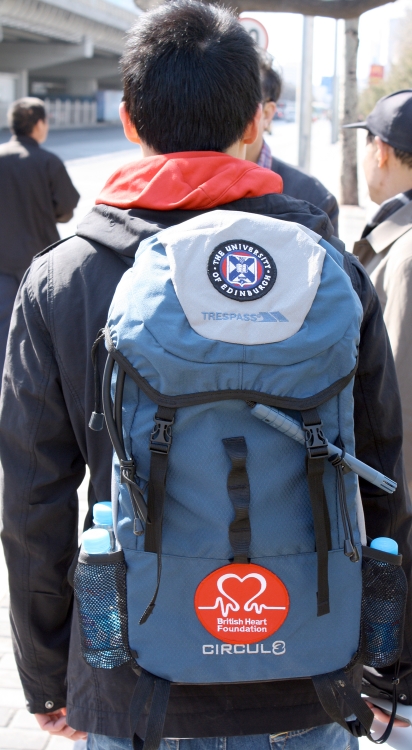
During their walks through the city center, subjects wore backpacks equipped with air monitors. Jeremy Langrish

The 98 participants that completed the study tolerated the face mask well and reported reductions in symptoms, perceived exertion, and perceived pollution levels. Use of a face mask was also significantly associated with beneficial cardiovascular characteristics, including reduced blood pressure and improved heart-rate variability.

The study was not double-blinded (in part because the researchers wanted to study the acceptability of wearing a face mask), and the findings may therefore be limited by subjective bias. The results suggest that face masks may effectively reduce individuals’ air pollutant exposure and lower associated cardiovascular risks. However, it is unknown whether these effects would be sustained with face-mask use over a longer period and would ultimately result in clinically significant improvement in cardiovascular health.

